# Cardiovascular-Kidney-Metabolic (CKM) Syndrome: A Case-Based Narrative Review

**DOI:** 10.1016/j.ajmo.2025.100089

**Published:** 2025-01-30

**Authors:** Aamir Javaid, Essa Hariri, Bige Ozkan, Katherine Lang, Sadiya S. Khan, Janani Rangaswami, Neil J. Stone, Roger S. Blumenthal, Chiadi E. Ndumele

**Affiliations:** a Division of Cardiology, Department of Medicine, Johns Hopkins University School of Medicine, Baltimore, Md.; b Division of Cardiology, Department of Medicine, Northwestern University Feinberg School of Medicine, Chicago, Ill.; c Division of Nephrology, Washington DC VA Medical Center; d George Washington University School of Medicine and Health Sciences, Washington, D.C.

**Keywords:** Cardiology, Nephrology, Prevention

## Abstract

These 4 hypothetical cases highlight new features of the American Heart Association cardiovascular-kidney-metabolic (CKM) health construct. The cases incorporate the CKM staging system, estimates from the PREVENT risk calculator, and clinical approaches related to CKM stages and individual risk profiles. Topics include management considerations for (1) a patient with stage 1 obesity and impaired glucose tolerance, (2) a patient with metabolic risk factors and moderate-risk chronic kidney disease (CKD), (3) a patient with subclinical atherosclerotic cardiovascular disease and multiple comorbid conditions, and (4) a patient with metabolic risk factors, prior myocardial infarction, new-onset heart failure, atrial fibrillation, and CKD.

**Clinical Significance.**

Cardiovascular-kidney-metabolic (CKM) syndrome is a novel construct which reflects the pathophysiological interplay of excess and/or dysfunctional adiposity, metabolic derangements, chronic kidney disease, and cardiovascular disease. The rising prevalence of obesity and diabetes across all age groups has resulted in poor CKM health in the population. A recent American Heart Association Presidential Advisory defines a new staging system for CKM syndrome that integrates novel risk prediction equations to both qualitatively and quantitatively stratify patients for total cardiovascular disease risk and determine optimal strategies for targeted interventions based on absolute risk and net benefit across each stage. The appropriate implementation of this guidance to address CKM-related risk, with an initial focus on lifestyle and then evidence-based medications and therapeutic approaches to reduce cardiovascular risk and kidney deterioration, will help improve CKM health and related outcomes in the population.

Alt-text: Unlabelled box

Cardiovascular-kidney-metabolic (CKM) syndrome is a novel construct which reflects the pathophysiological interplay of excess and/or dysfunctional adiposity, metabolic derangements, chronic kidney disease (CKD), and cardiovascular disease (CVD). The rising prevalence of obesity and diabetes across all age groups has resulted in worsening CKM health in the population. A recent American Heart Association (AHA) Presidential Advisory defines a new staging system for CKM syndrome that integrates novel risk prediction equations to both qualitatively and quantitatively stratify patients for total CVD risk and determine optimal strategies for targeted interventions based on absolute risk and net benefit across each stage.^1^^,^^2^

Earlier identification of individuals on the CKM syndrome spectrum will help address obesity as an upstream treatment target using a holistic approach, guide cardio-kidney-metabolic protective pharmacotherapies to reduce CVD risk, identify social and structural barriers to care, prevent progression to more advanced disease, and facilitate regression to an earlier stage along the CKM spectrum. This article did not involve human subjects and is exempt from Institutional Review Board approval. The following four hypothetical cases from each of the CKM stages were selected to illustrate key management considerations based on the synopsis of available guidance from the new AHA CKM Presidential Advisory.

## Case 1

A 57-year-old female with no significant past medical history presents to primary care for an annual physical. She has attempted to lose weight for the past 3 years unsuccessfully. She has tried multiple diets, including Keto and Paleo diets, and tries to cook healthy meals at home. She walks her dog every day for 45 minutes in her neighborhood. She is a former tobacco smoker who quit more than 25 years ago. Her family history is pertinent for diabetes in her mother and stroke in her father. She usually sleeps 8 hours a night but is fatigued during the day and does not feel she gets refreshing sleep even on the weekends. Her husband told her she snores. Her physical exam reveals height 5 feet 2 inches (157.5 cm), weight 165 lbs (75 kg), body mass index [BMI] 30.2 kg/m^2^, waist circumference 33 inches (84 cm), and BP 124/74 mm Hg. Blood tests indicate fasting glucose of 110 mg/dL, creatinine of 0.7 (estimated glomerular filtration rate [eGFR] 103 mL/min/1.73 m^2^), A1c 5.5 %, thyroid stimulating hormone (TSH) 1.4 mU/L, total cholesterol (TC) 170 mg/dL, triglycerides (TG) 120 mg/dL, high density lipoprotein cholesterol (HDL-C) 52 mg/dL, and low-density lipoprotein cholesterol (LDL-C) 110 mg/dL.

### Assessment

This patient is categorized as CKM stage 1, characterized by excess body fat and/or an unhealthy distribution of body fat in the absence of overt metabolic risk factors or CKD. The framework for targeted risk-based prevention using CKM stages begins with screening guided by Life's Essential 8.^3^ This includes assessment of dietary patterns, physical activity, sleep duration and quality, nicotine exposure, body mass index, blood pressure, lipids, and blood sugar. Additional testing can be considered as clinically indicated, including hemoglobin A1c for those with diabetes or overweight/obesity (in this case, A1c was checked given her obesity and family history of diabetes) and urine albumin to creatinine ratio (UACR) for those with CKM stage 2 and above.^2^ Among adults aged 30 to 79 years, the 10- and 30-year absolute risk of CVD (a composite of atherosclerotic cardiovascular disease [ASCVD] and heart failure [HF]), as well as ASCVD and HF individually can be calculated with the PREVENT score.^2^ This patient's 10-year risk estimates for total CVD, ASCVD, and HF are 3%, 2%, 1%, and 30-year risk estimates are 20%, 11%, and 10%, respectively. For comparison, the 10-year risk for ASCVD when estimated with the pooled cohort equation (PCE) is 2%, and the lifetime risk is 27%.^4^

The CKM framework and PREVENT risk calculator were developed, in part, to account for changes in risk factor prevalence (rising rates of obesity and CKD, declining tobacco use, rising triglycerides, and lower LDL-C levels)^5^ and care patterns (eg, more widespread use of antihypertensive and lipid-lowering therapies) in the United States. The PREVENT score also helps to further personalize risk estimates through incorporation of markers of kidney, metabolic, and social factors, accounting for the interrelated and upstream effects of CKM conditions on CVD risk. This may also provide a framework for considering novel therapies that address all 3 axes of CKM disease, with management considerations discussed in the CKM guidance and the present article.^2^

Although 10-year risk estimates are low, she is at elevated lifetime risk, which can be reduced through management of her CKM risk factors. Her BMI indicates stage 1 obesity. Her waist circumference reflects central adiposity, with cut points of ≥88 cm (35 inches) in women and ≥102 cm (40 inches) in men and suggested lower thresholds of 80 (31.5 inches) and 90 cm (35.5 inches) for women and men of Asian ancestry.^6^

While normal fasting glucose is less than 100 mg/dL, her glucose value of 110 mg/dL indicates impaired glucose tolerance, although her A1c of 5.5 % is below the cut-off of 5.7% for prediabetes. The cut-offs for diabetes are a fasting glucose of ≥126 mg/dL or A1c ≥6.5% on repeated measurements.^7^ While A1c has several advantages over fasting glucose, such as convenience (patients do not have to be fasting), decreased day-to-day variability, and greater preanalytic stability, it is not that sensitive or specific for diagnosing prediabetes, while fasting glucose is specific but not sensitive.^8^ The oral glucose tolerance test, in comparison, has the greatest sensitivity for detecting impaired glucose tolerance and can be considered for this patient with multiple risk factors for metabolic syndrome and progression to overt diabetes, but fasting glucose and A1c are typically preferred because of the ease of administration.^9^

### Management

For asymptomatic adults with stage 1 CKM, screening a minimum of every 2 to 3 years is advised to reassess blood pressure, lipid profile, and blood sugar, with more frequent visits recommended for patients with abnormal biomarkers, such as elevated fasting glucose in this case, based on patient-clinician preference. Nonjudgmental weight loss counseling increases the likelihood of successful weight loss attempts. The STOP (Strategies To Overcome and Prevent) Obesity alliance toolkit provides a framework for these discussions.^10^^,^^11^ Comprehensive lifestyle intervention may be effective for sustained weight loss. This includes a behavior change program to support chronic maintenance of a net caloric deficit through moderate caloric restriction with emphasis on diets with strong evidence in cardiovascular risk reduction, such as the Mediterranean or Dietary Approaches to Stop Hypertension (DASH) diets, and at least 150 minutes of moderate or 75 minutes of vigorous aerobic activity per week.^4^

At least 5% weight loss for individuals with CKM stage 1 is suggested for beneficial effects on cardiometabolic risk factors. Greater weight loss of 10% of body weight is associated with more significant improvements in BP, glycemia, and lipids, while ≥10% weight loss may be associated with lower CVD event rates.^1^ Given her reports of fatigue, diurnal sleepiness, and snoring, she should be evaluated for sleep-disordered breathing with polysomnography and prescribed continuous positive airway pressure therapy if she is diagnosed with obstructive sleep apnea.

Lastly, addressing social determinants of health (SDOH) has a prominent impact on all stages of CKM. The PREVENT model has the option to include the social deprivation index at the zip code level. However, despite interest in inclusion of measures that more directly reflect risk-related additional individual- and place-based measures of social drivers (eg, racism, income, education, residential green space), the lack of standardized assessment and capture in data sources was a key limitation. Although this is an important first step to factor social drivers of risk in risk assessments, numerous additional screening tools exist to quantify the social, environmental, and psychosocial factors that affect health, which are detailed in the AHA Presidential Advisory.^1^ When adverse SDOH are identified, it is important to have individuals integrated within the care team that can help to address them, such as community health workers, social workers, or care navigators.

## Case 2

A 60-year-old White male with a history of hypertension, obesity (BMI 32 kg/m^2^) and prediabetes presents to the primary care clinic for routine examination after several years. He is not taking any medications. He has never used tobacco products. His exam is notable for blood pressure 146/84 mmHg, heart rate 88, and weight 245 lbs (BMI 32 kg/m^2^). His waist circumference is 41 inches (105 cm). Fasting lab work indicates: creatinine 1.2 mg/dL (eGFR 68 mL/min/1.73 m^2^), glucose 136 mg/dL, HbA1c 7.9%, total cholesterol 207 mg/dL, triglycerides 178 mg/dL, HDL-C 35 mg/dL, LDL-C 141 mg/dL, urine albumin/creatinine ratio (UACR) 45 mg/g (normal < 30 mg/g).

### Assessment

This patient has stage 2 CKM syndrome, which is characterized by metabolic risk factors and/or moderate or high-risk CKD. He meets these criteria based on stage 2 hypertension (systolic BP ≥140 mm Hg), metabolic syndrome (abdominal obesity with abdominal circumference ≥102 cm, triglycerides >150 mg/dL, HDL-C < 40 mg/dL, hypertension, and diabetes) and moderate risk CKD using the Kidney Disease: Improving Global outcomes (KDIGO) risk heat map for adverse kidney and cardiovascular disease outcomes, as reflected by a mildly decreased eGFR (60-89 cc/min/1.73 m^2^, G2) and moderately increased albuminuria (UACR 30-300 mg/g, A2).^12^ His PREVENT 10-year risk estimates for total CVD, ASCVD, and HF are 20%, 13%, 11%, respectively.^2^ The risks for ASCVD and HF do not add numerically to total CVD risk, because risk estimates for each CVD subtype are based on the total incidence of events for that subtype, and each score is from a different risk estimation model. For comparison, his 10-year risk for ASCVD by the PCE is 14%.^4^ There are no 30-year estimates for patients 60 years old or older.

Compared to the PCE, the PREVENT score has significantly improved discrimination and calibration, particularly at higher risks. The PCE was noted to overestimate risk when tested in modern cohorts.^13^ This is reflected by the slightly lower predicted risk of ASCVD using PREVENT in the current example. Furthermore, this case illustrates the significance of accounting for this patient's CKD and obesity to estimate the total risk of CVD, which is notably ≥20%. Although specific cutpoints using the PREVENT score have not been established for management decisions, comprehensive assessment using PREVENT underscores the importance of improved management along the entire spectrum of his interrelated CKM risk profile. The challenge with all risk calculators is the potential for imprecision; however, as we illustrate in the following section, the PREVENT score can serve as the foundation for patient-clinician conversations to aid decisions regarding in whom, when, and how urgently to initiate therapies.

### Management

The goal for patients in CKM stage 2 is to treat metabolic risk factors and CKD and prevent progression to subclinical and clinical CVD. Therefore, this patient requires intervention for his diabetes, hypertension, obesity, dyslipidemia, and CKD. Lifestyle changes, with pharmacotherapy as needed for risk factor control, should be supported to improve cardiovascular outcomes. Lifestyle change can often lower triglycerides to <150 mg/dL. This patient should be treated to a goal HbA1c <7%, blood pressure <130/80 mmHg, and with lipid-lowering therapy to facilitate at least moderate (30%-50%) or marked (50%) reduction in his LDL-C, consistent with the CKM Advisory and the 2018 AHA/American College of Cardiology (ACC)/Multi-society Cholesterol Guideline.^1^^,^^14^

For his cholesterol, a moderate- to high-intensity statin is appropriate. Use of a renin-angiotensin-aldosterone-system (RAAS) inhibitor, such as an angiotensin receptor blocker (ARB), should be part of the antihypertensive regimen for patients with diabetes with albuminuria or patients with CKD for additional protection of kidney function. Although this patient had a single urine sample with albuminuria >30 mg/g, a second sample is usually recommended to confirm albuminuria.

For patients with diabetes, when considering cardio-kidney protective anti-hyperglycemic agents, sodium-glucose cotransporter-2 inhibitors (SGLT2i) should be prioritized for those with CKD, given their favorable impact on kidney function decline, as well as the risks of HF and cardiovascular mortality.^15^ Glucagon-like-peptide-1 receptor agonists (GLP-1RA), the other major class of cardioprotective antihyperglycemic agents, would also benefit this patient to treat his obesity, diabetes, and reduce risk of major adverse cardiovascular events and major kidney disease events, as demonstrated in the FLOW trial.^16^ The combination of SGLT2i and GLP-1RA may result in complementary benefits, given the confluence of CKM factors and the high total CVD risk, which is also supported by KDIGO CKD 2024,^17^ although randomized control trial evidence for dual use is not yet published. In HARMONY Outcomes and AMPLITUDE-O, SGLT2i was not an effect modifier for clinical benefit from GLP-1RA, but baseline SGLT2i use was infrequent in both trials.^18^^,^^19^ The choice of which agent to start first may be guided by several factors—including risk factor profile (such as more evidence of benefit for SGLT2i in heart failure and chronic kidney disease and more benefit of GLP-1RA to treat obesity), cost, and patient preference—with upfront combination use also reasonable. A meta-analysis found there is evidence of increased risk of side effects, such as GI upset and injection site-related events, with the addition of GLP-1RA to SGLT2i, but no increase in serious adverse or cardiovascular events.^20^

Metformin co-utilization with an SGLT2i can also be considered for those with HbA1c ≥7.5% to help achieve glycemic targets with minimal side effects and high affordability. This is important because SGLT2i, while having a potent impact on preventing cardiovascular and kidney events, has only a modest impact on glycemia (average decrease in A1c of 0.5%-1%). Therefore, given this patient's A1c of 7.9%, the addition of metformin to an SGLT2i should be considered to control hyperglycemia, particularly if GLP-1RA is cost-prohibitive. Metformin can be begun at 500 mg daily and uptitrated to 1000 mg BID as needed, if tolerated. Additionally, with a confluence of diabetes and CKD, a CKM care coordinator or advanced practice provider would be helpful to facilitate interdisciplinary patient-centered care by aiding the stepwise addition of and adherence to therapies, assessing for adverse SDoH, and providing longitudinal support between visits to favorably impact metabolic risk factors and kidney disease and reduce cardiovascular risk in the long term.

## Case 3

A 52-year-old Black male presents to clinic for post-hospital discharge follow-up after a pneumonia. He is feeling back to baseline with no chest pain or shortness of breath. He has a history of obesity (BMI 35 kg/m^2^), hypertension (controlled on losartan 100 mg and amlodipine 10 mg), untreated diabetes, and CKD stage 3 (G3aA2) (eGFR 50 mL/min/1.73 m^2^, UACR of 80 mg/g). He has never smoked. His BP is 125/85 and his waist circumference is 126 cm (49.6 inches). Recent fasting lipid panel showed TC 210 mg/dL, TG 180 mg/dL, HDL-C 40 mg/dL, LDL-C 135 mg/dL, and his A1c is 9.2%. A noncontrast CT of the chest was performed in the inpatient setting, which revealed a coronary artery calcium (CAC) score of 50 (88th percentile for age, sex, and race and ethnicity).^21^

### Assessment

This patient has CKM stage 3—subclinical cardiovascular disease without symptoms—given the presence of CAC >0. His 10-year CVD risk by the PREVENT equation is 21%, and he has an estimated 12% risk of ASCVD and 15% risk of HF. The 30-year risks are 49%, 29%, and 42%, respectively. This is compared to an estimated 18% 10-year risk of ASCVD and 69% lifetime risk by the PCE.^2^^,^^4^ His high PREVENT risk estimate for total CVD reflects the need for more intensive, comprehensive management of the patient's CKM risk profile. The 10-year risk of heart failure is comparable to the Pooled Cohort Equations to Prevent HF (PCP-HF) score of 15%.^22^ The PREVENT score has another advantage of saving user time by calculating these risks with one set of model inputs, versus what previously required multiple calculators.

### Management

This patient has class II obesity (BMI 35 to <40 kg/m^2^), uncontrolled diabetes, CKDG3aA2, and subclinical ASCVD. The use of UACR helps to further classify his CKD stage, with albuminuria categories of <30 mg/g for stage 1 (normal to mildly increased), 30-300 mg/g for stage 2 (moderately increased), and >300 mg/g for stage 3 (severely increased). Higher stages are associated with greater risks of kidney and major adverse cardiovascular events.^17^ Albuminuria is a powerful biomarker for adverse kidney and cardiovascular outcomes,^23^ but albuminuria quantification is significantly underutilized, even in patients with established CKD, as shown in recent data from the CURE-CKD registry.^24^ He would benefit from a GLP-1RA to treat his obesity, diabetes, and reduce risk of major adverse cardiovascular events, as demonstrated in the FLOW trial.^16^ Similarly, both ACEi/ARB and SGLT2i in CKD with eGFR >20 mL/min/1.73 m^2^ are protective of kidney function and against major adverse cardiovascular events. As discussed in the previous case, the combination of SGLT2i and GLP-1RA may result in complementary benefits, given the confluence of CKM factors and the high absolute CVD risk associated with stage 3 CKM. Given the patient's obesity, GLP-1RA can be argued as the best first choice with SGLT2i added subsequently as a class 1A indication in a patient with G3aA2 CKD. If cost for a GLP-1RA is prohibitive, metformin is also a reasonable choice to help achieve glycemic targets with less financial burden for patients, but metformin likely will not provide comparable cardioprotective benefits. Given he is treatment naive, these agents should be trialed before insulin initiation, which is typically initiated for severe hyperglycemia (blood glucose >300 mg/dL), A1c >10%, or symptoms of hyperglycemia, such as polyuria or polydipsia, or evidence of catabolism, including unexpected weight loss.^25^ Higher A1c values are associated with higher incidence of adverse side effects with SGLT2i, such as genitourinary infections and euglycemic diabetic ketoacidosis.^26^ A CKM coordinator, likely a physician assistant, nurse practitioner, or diabetes health educator, would be helpful with implementing therapies to address multisystem risk in this case and to help uptitrate GLP-1RA to the maximal tolerated dose.

The presence of mild (but above average for his age) CAC indicates subclinical atherosclerosis. Given his intermediate 10-year risk of ASCVD, initiation of statin therapy should be strongly considered for absolute risk reduction for ASCVD events.^4^

## Case 4

A 68-year-old male presents to the emergency department with several days of progressive dyspnea. His history includes hypertension, hyperlipidemia, grade I obesity (BMI 32 kg/m^2^), diabetes (HbA1c 7.3% 3 months prior), former smoking (20-pack year history, quit 5 years prior), CKD 3A (baseline Cr 1.4 mg/dL, eGFR 55 mL/min/1.73 m^2^), prior myocardial infarction (s/p percutaneous coronary intervention with 2 drug-eluting stents to the left anterior descending artery 7 months prior). His chronic medications include metoprolol, atorvastatin, aspirin, clopidogrel, and metformin.

On exam, his vital signs are heart rate 112 bpm, blood pressure 126/78 mm Hg, respiratory rate 24 breaths/min, and O_2_ saturation 87% on room air. Cardiovascular exam reveals tachycardia that is irregularly irregular, S3, no murmurs, and JVP measured at 13 cm H_2_O above the RA. Lung auscultation reveals rales to the mid-lung fields. His bilateral lower extremities are warm and have 2+ pitting edema to the mid-shins.

His labs reveal NT-proBNP 13,909 pg/mL, hs-troponin <5 ng/L × 3, lactate 1.7 mmol/L, BUN 29 mg/dL, Cr 1.7 mg/dL (eGFR 43 mL/min/1.73 m^2^), TC 140 mg/dL, TG 103 mg/dL, HDL-C 40 mg/dL, and LDL-C 85 mg/dL. Echocardiography reveals an ejection fraction of 35%-40%. He is admitted for management of acute decompensated heart failure and atrial fibrillation. He is successfully diuresed and started on guideline-directed medical therapies (GDMT) for HFrEF, including sacubitril-valsartan, spironolactone, and metoprolol. His vitals and exams improve significantly throughout his hospital course. He is started on apixaban, and aspirin is discontinued in the setting of atrial fibrillation. His lab work remains stable, and his kidney function returns to baseline.

### Assessment

This patient meets criteria for stage 4 CKM, which is characterized by symptomatic/clinical CVD, as evidenced by his known coronary disease with prior MI, new-onset heart failure, atrial fibrillation, and concomitant metabolic risk factors (hypertension, type 2 diabetes, obesity), and stage 3a CKD. The goal of management for patients with stage 4 CKM centers around optimizing metabolic risk factors, kidney function, and existing CVD by focusing on comprehensive secondary prevention. As such, this patient has several comorbidities that should be addressed as follows.

### Management

For this patient with grade I obesity, both BMI and waist circumference should be measured at least annually for assessing adiposity-related risk. The presence of abdominal obesity, particularly in the setting of metabolic syndrome, strongly indicates a need for lifestyle change and weight loss, with a discussion that can be introduced using the STOP obesity toolkit, as mentioned previously. Particularly for those in higher CKM syndrome stages, weight loss should be supported by an integrated multidisciplinary team to facilitate intensive lifestyle modification and access to adjunctive weight loss therapies, as needed.

As for many patients with stage 4 CKM, this patient has very high-risk CHD, given the presence of a prior acute MI, multiple comorbidities, including diabetes, hypertension, CKD and HF, and age ≥65 years. Therefore, additional lipid-lowering therapy beyond statin therapy should be considered in the setting of an LDL-C ≥70 mg/dL, as per the 2018 AHA/ACC/Multi-society Cholesterol Guidelines and the CKM Presidential Advisory. The addition of generic ezetimibe should be the first-line treatment, which is adequate for controlling LDL-C for most patients, with the subsequent additional evidence-based LDL-C-lowering agents in the future, if needed.

For his atrial fibrillation, multiple CKM factors are linked to a greater likelihood and burden of atrial fibrillation, as well as a higher risk of stroke in those with atrial fibrillation, including ASCVD, hypertension, diabetes, obesity, and CKD. Therefore, in addition to anticoagulation for those with elevated stroke risk, major guidelines recommend comprehensive risk factor control in patients with atrial fibrillation.^27^ Furthermore, weight loss, regular physical activity, and improved cardiorespiratory fitness are advised to decrease atrial fibrillation burden and severity in patients with CKM syndrome. An outpatient sleep study to screen for obstructive sleep apnea should be performed in the context of obesity and atrial fibrillation. If present, appropriate treatment of obstructive sleep apnea may also reduce atrial fibrillation burden.^27^

For his heart failure with reduced ejection fraction (HFrEF), he should be initiated on all four pillars of GDMT. He is already taking an angiotensin receptor/neprilysin inhibitor (ARNi), a mineralocorticoid receptor antagonist (MRA), and a beta blocker. An SGLT2i should be the next priority for further HFrEF optimization in this setting. While hydralazine/isosorbide dinitrate also has mortality benefits in HFrEF for Black patients, this would not be prioritized before SGLT2i.^28^

He has moderate-risk CKD based on his eGFR and needs an assessment of UACR, when in steady state in the ambulatory setting, for further characterization and risk stratification of CKD risk. Because of his CKD, the use of cardio-kidney protective agents is indicated. In this case, the use of an SGLT2i will improve his heart failure outcome and prevent kidney function decline, so it is indicated for both reasons. Finerenone is also associated with lower risks of CKD progression in diabetes, with associated cardiovascular benefits,^29^ but an SGLT2i should be the frontline kidney protective agent in the setting of HF. Furthermore, based on current evidence, the steroidal MRA eplerenone (if intolerant of spironolactone) would be favored in the setting of HFrEF over the nonsteroidal MRA finerenone.^30^

In the setting of suboptimally controlled diabetes and CVD, it is important to add an SGLT2i and/or a GLP-1RA as a cardioprotective antihyperglycemic agent. In this patient with HF and CKD, the use of an SGLT2i is the highest priority. However, incretin analogs, such as GLP-1Ras, induce marked intentional weight loss and improve glycemia and metabolic risk factor control. The recent SELECT (Semaglutide Effects on Heart Disease and Stroke in Patients With Overweight or Obesity) clinical trial showed that high-dose GLP-1RAs reduce cardiovascular events in patients with overweight/obesity and CVD, even in the absence of diabetes.^31^ In this case, the patient has high CVD risk due to prevalent CVD and multiple comorbidities, which might favor the use of GLP-1RA in addition to SGLT2i. However, there are some caveats. The SELECT trial excluded individuals with acute cardiovascular events in the last 60 days, so the current evidence supports the initiation of high-dose GLP-1RA in more stable CVD patients with overweight/obesity. The indications for dual use of SGLT2i and GLP-1RA are not yet well defined but could be considered for those with multiple comorbidities in the setting of diabetes and CVD. The 2023 European Society of Cardiology Guidelines for the management of cardiovascular disease in patients with diabetes recently recommended concomitant use in high-risk patients.^32^

From a metabolic standpoint, his A1c is near goal, and his BMI is not severely elevated, which somewhat reduces the urgency of GLP-1RA use. However, the SELECT trial found similar benefits of high-dose semaglutide on cardiovascular events in those with BMI ≥30 kg/m^2^ and those with BMI 27-30 kg/m^2^. Additionally, although there is growing data about the benefits of GLP-1RA in HFpEF,^33^ and the SELECT trial indicated significant cardiovascular risk reduction in the subgroup with heart failure, there is not yet definitive data regarding their primary utilization as foundational therapy in HFrEF. Therefore, while combination therapy with cardioprotective antihyperglycemics (SGLT2i and GLP-1RA) might be considered, the considerations described above somewhat weaken the indications for upfront GLP-1RA use in this patient with prevailing data.

Once again, for this patient with a confluence of CVD, diabetes, and CKD, a CKM coordinator can play an important role in facilitating adherence with multiple therapies to reduce CKM risk, as well as in supporting patient navigation across multiple subspecialists.

## Conclusion

The CKM health construct represents a novel paradigm for cardiovascular prevention and management that reflects the rising prevalence and interplay among metabolic disease, CKD, and CVD. In this article, we have detailed 4 patient vignettes representing each of the CKM stages with commentary on how the CKM guidance can help improve management of these patients’ conditions. Given the growing number of therapies that simultaneously address all axes of CKM syndrome, the CKM framework helps identify at-risk individuals and select interventions to prevent disease progression and, in some cases, promote regression to earlier CKM stages. In addition, the CKM algorithms also provide guidance for approaching management for the patient with CVD overlapping with metabolic risk factors, CKD, or both. The appropriate implementation of this guidance to address CKM-related risk, with an initial focus on lifestyle and then evidence-based medications and therapeutic approaches to reduce cardiovascular risk and kidney deterioration, will help improve CKM health and related outcomes in the population.

## CRediT authorship contribution statement

**Aamir Javaid:** Writing – review & editing, Writing – original draft, Conceptualization. **Essa Hariri:** Writing – review & editing, Writing – original draft. **Bige Ozkan:** Writing – review & editing, Writing – original draft. **Katherine Lang:** Writing – review & editing, Writing – original draft. **Sadiya S. Khan:** Writing – review & editing. **Janani Rangaswami:** Writing – review & editing. **Neil J. Stone:** Writing – review & editing. **Roger S. Blumenthal:** Writing – review & editing. **Chiadi E. Ndumele:** Writing – review & editing, Conceptualization.

## Declaration of competing interest

The authors declare that they have no known competing financial interests or personal relationships that could have appeared to influence the work reported in this paper.
